# Caries lesions diagnosis with deep convolutional neural network in intraoral QLF images by handheld device

**DOI:** 10.1186/s12903-024-04517-x

**Published:** 2024-06-29

**Authors:** Rukeng Tan, Xinyu Zhu, Sishi Chen, Jie Zhang, Zhixin Liu, Zhengshi Li, Hang Fan, Xi Wang, Le Yang

**Affiliations:** 1 Guanghua School of Stomatology, Hospital of Stomatology, Sun Yat-sen University, 56th Lingyuanxi Road, Guangzhou, 510055 Guangdong China; 2 Guangdong Province Key Laboratory of Stomatology, No. 74, 2nd Zhongshan Road, Guangzhou, 510080 Guangdong China; 3 Guangzhou Stars Pulse Co., Ltd, 239th Tianhe North Road, Tianhe District, Guangzhou, 510610 Guangdong China

**Keywords:** Caries, Quantitative light-induced fluorescence, Artificial intelligence, Handheld device, Home use

## Abstract

**Objectives:**

This study investigated the effectiveness of a deep convolutional neural network (CNN) in diagnosing and staging caries lesions in quantitative light-induced fluorescence (QLF) images taken by a self-manufactured handheld device.

**Methods:**

A small toothbrush-like device consisting of a 400 nm UV light-emitting lamp with a 470 nm filter was manufactured for intraoral imaging. A total of 133 cases with 9,478 QLF images of teeth were included for caries lesion evaluation using a CNN model. The database was divided into development, validation, and testing cohorts at a 7:2:1 ratio. The accuracy, sensitivity, specificity, positive predictive value, negative predictive value, and area under the receiver operating characteristic curve (AUC) were calculated for model performance.

**Results:**

The overall caries prevalence was 19.59%. The CNN model achieved an AUC of 0.88, an accuracy of 0.88, a specificity of 0.94, and a sensitivity of 0.64 in the validation cohort. They achieved an overall accuracy of 0.92, a sensitivity of 0.95 and a specificity of 0.55 in the testing cohort. The model can distinguish different stages of caries well, with the best performance in detecting deep caries followed by intermediate and superficial lesions.

**Conclusions:**

Caries lesions have typical characteristics in QLF images and can be detected by CNNs. A QLF-based device with CNNs can assist in caries screening in the clinic or at home.

**Trial registration:**

The clinical trial was registered in the Chinese Clinical Trial Registry (No. ChiCTR2300073487, Date: 12/07/2023).

**Supplementary Information:**

The online version contains supplementary material available at 10.1186/s12903-024-04517-x.

## Introduction

Currently, caries detection depends on a doctor’s visual-tactile inspection; the International Caries Detection and Assessment System (ICDAS) is the most widely used visual classification system [1]. As the most common oral disease, caries is a chronic bacterium-infected disease that invades enamel and dentin to the pulp, graded on a scale of 0 to 6 in the ICDAS. If necessary, radiology is performed to assist in diagnosis. It has been reported that periapical and bitewing films are more effective at detecting adjacent and occult caries but have low sensitivities, ranging from 0.24 to 0.42 [2]. However, the validity of these examinations varies among individuals, and it is difficult to detect early demineralization symptoms of caries.

Timely identification of caries is important for preserving diverse oral tissues and reducing personal and public financial burdens. However, this approach is limited by public attention to dental health and the experience of doctors, which are affected by both subjective and objective factors [3]. Currently, there are an increasing number of leading assistive technologies for caries detection, such as optical coherence tomography, QLF, and fibre optic transillumination imaging, based on physical or chemical characteristics [4–6]. Although these methods can achieve overall accuracies ranging from 0.72 to 0.91 with average repeatability, some limitations remain [2, 7–10]. Specific changes in the colour and texture caused by plaque, white spots, and others exhibiting similar physical characteristics may cause confusing results. Moreover, the judgement of experienced doctors is still needed, and the devices are usually large, preventing their flexible clinical use.

QLF is a technique that uses a blue light source to illuminate the tooth surface and reflects oral problems through differences in fluorescence intensity in the irradiated area. It works by detecting and quantifying the differences between spontaneous enamel fluorescence and fluorescence loss associated with the demineralized region, which is directly related to the enamel’s mineral content. It can distinguish healthy enamel and caries [11]. Using the QLF technique to evaluate the demineralization of teeth in clinical trials and related studies has greatly reduced the work of researchers [2]. However, QLF accuracy is not as good at diagnosing proximal caries, and it still depends on the subjective identification by doctors. CNNs are an important type of artificial intelligence (AI) for automatically identifying the hierarchical characteristics of data through algorithm learning. Introducing CNNs can effectively eliminate subjective factors in the diagnosis of dental caries [12]. Due to its excellent image recognition ability, CNNs have been used to evaluate tooth defects, periodontal alveolar bone loss, and other problems [13]. Within this context, YOLOv5s, as a CNN model, stands out for its efficiency and speed. You only look once (YOLO), which is renowned for its widespread adoption in real-time object detection tasks, can swiftly and accurately localize and categorize multiple objects in both images and videos [14]. This distinctive feature positions YOLOv5s as an optimal choice, particularly for deployment on fast and efficient mobile devices. Consequently, YOLOv5s presents a dual advantages in terms of practicality and precision for dental image processing in dentistry [15, 16].

Here, we fabricated a small camera that emits a specific wavelength of light as an input device to detect caries. YOLO v5 was used as a CNN network for model development. Finally, we combined the images of the teeth with diagnosis points as an output and evaluated the model performance.

## Materials and methods

### Study design

This study was approved by the Institutional Review Board of the Hospital of Stomatology, Sun Yat-sen University, and was conducted in accordance with the Declaration of Helsinki (KQEC-2023-32-02). This study was registered in the Chinese Clinical Trial Registry (ChiCTR2300073487).

**Fig. 1:**
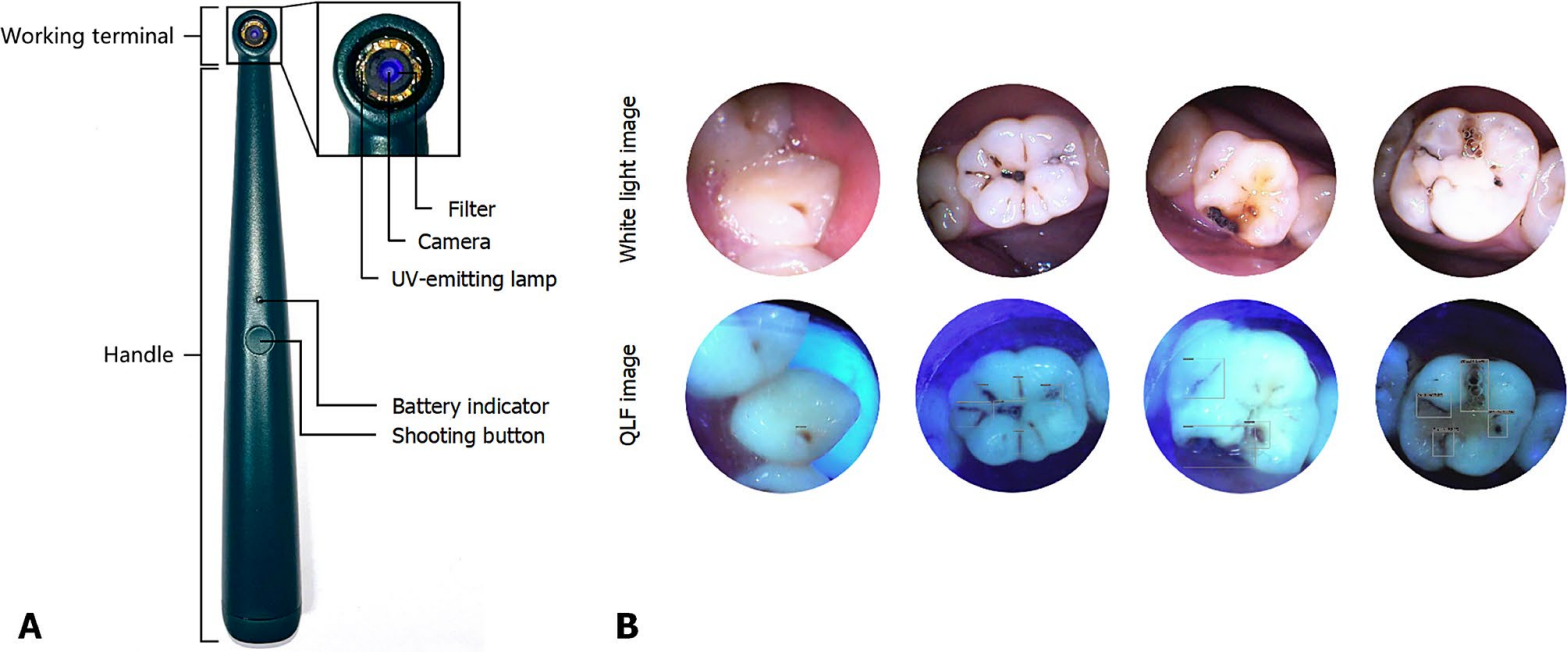
QLF-based handheld device and its typical images. (**A**) External view of the QLF-based handheld device; B. Typical QLF images based on the ICDAS. (**B**) Notes: The boxes shown on the QLF images were automatically real-time generated by the caries detection model

**Fig. 2:**
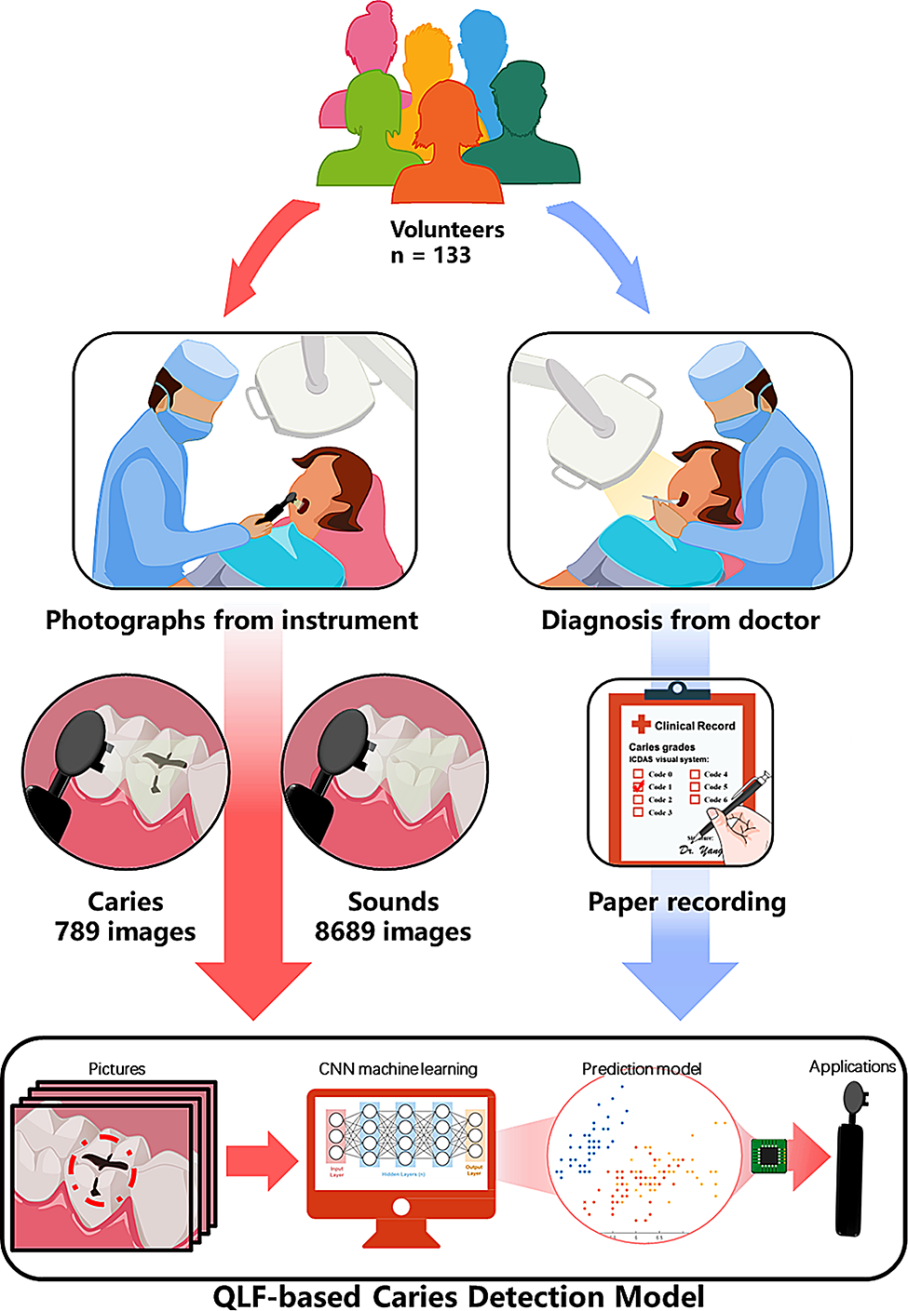
Flowchart of the study design

Before recruiting volunteers for this research, we manufactured a small and convenient QLF image capture device based on the QLF principle (Fig. 1). The device, which is shaped like a toothbrush, includes a 400 nm UV light-emitting lamp, a mini camera, a 470 nm filter, and a receiver. The width of the device’s head is only 1 cm, making it very easy to collect images of teeth from different angles inside the oral cavity. Volunteers were informed about the acquisition and use of all the data and signed the informed consent form. All of the recruited volunteers agreed to participate except those who were undergoing orthodontic treatment, could not complete the examination and sampling procedures, and did not agree to participate in the project. Basic information such as sex, age, and oral hygiene habits of the volunteers was collected. First, we obtained QLF images according to established shooting standards from our original database. Then, the endodontists manually bound and labelled caries lesions in the images based on the ICDAS-II criteria. After that, YOLOv5s, an algorithm based on deep neural networks, was applied to create a model that can detect and stage caries automatically in QLF images. The study flowchart is shown in Fig. 2.

### Dataset

A total of 9478 QLF images of teeth on various surfaces from 133 volunteers were obtained at the Hospital of Stomatology, Sun Yat-sen University, between August 2023 and September 2023. In addition, two experienced endodontists examined each tooth of the volunteer according to the ICDAS-II criteria [1, 2], which were also recorded and analysed. The consistency of the results of the two dentists’ examinations were recorded. In cases where the results were inconsistent, the two endodontists discussed and verified the results until they reached a consensus, which was recorded. They also needed to mark the location of the caries (ICDAS score > 0) on the record sheet. Those with caries were classified as superficial (ICDAS score of 1 or 2), intermediate (ICDAS score of 3 or 4) or deep (ICDAS score of 5 or 6). We used the same equipment to obtain all the QLF fluorescence images according to uniform standards. Every tooth except the third molar and the missing tooth were photographed and placed in the centre of the image. The dataset was split as follows: 70% for training (6634 QLF images), 20% for validation (1896 QLF images), and 10% for testing (948 QLF images). The testing set was never made available as training material and served as an independent set. We use the QLF images directly for training without any enhancement or preprocessing methods. The 9478 QLF images were augmented four times via rotation, scaling, zooming, and cropping operations.

### Data labelling

The caries lesions in the 9478 QLF images in this study were labelled by two expert dentists according to the recorded lesion location. In the labelling process, an irregular box was drawn according to the outline of each caries lesion for the YOLOv5s training process. The labelling process was performed using labelImg.exe, which is programmed in Python.

### Model training and data analysis

We used YOLOv5s as our training core algorithm. The training of the CNN was repeated 3 times. The resulting model was evaluated on the validation and testing cohorts. The diagnostic accuracy (ACC), sensitivity (SEN), specificity (SPE), positive predictive value (PPV), negative predictive value (NPV), F1-score, precision-recall curve (PRC), and area under the receiver operating characteristic (ROC) curve (AUC) were estimated. *P* values < 0.05 were considered to indicate statistical significance, and 95% confidence intervals (CIs) were calculated.

## Results

There were 133 participants, 3690 of whom were photographed. Among the participants, males accounted for 24.82% and females accounted for 75.19%, with a male‒female ratio of 0.33. Females had a greater average oral hygiene habit score than males did (average score: female, 1.82; male, 1.61). The mean decayed, missing and filled teeth (DMFT) and mean PLI of all participants were 6.71 and 1.18, respectively. The characteristics of all the subjects are shown in Table 1. Of all the teeth captured in the images, 1592 were anterior teeth, 1037 were premolars, and 1061 were molars. In total, 75.69% of the total teeth were sound, 19.59% had caries, and 4.69% had fillings. Molars had the highest percentage of caries and fillings, at 48.92% and 13.38%, respectively. Compared with the other two tooth surfaces, the occlusal surface had the most caries (23.93%). The detailed data are shown in Table 2.

**Table 1:** Characteristics of the study cases

Characteristics	Number (%)		
	Male	Female	Total
Number of cases	33 (24.82%)	100 (75.19%)	133 (100%)
Number of teeth	921 (24.96%)	2769 (75.04%)	3690 (100%)
Age^^^	17–25	18–27	17–27
**Oral Hygiene Habit**			
1	14	27	41
2	18	64	82
3	1	9	10
Average score^#^	1.61 ± 0.56	1.82 ± 0.58	1.77 ± 0.58
**DMFT**			
Sound	724 (78.61%)	2068 (74.68%)	2793 (75.69%)
Decay	186 (20.2%)	537 (19.39%)	723 (19.59%)
Missing	9 (0.98%)	36 (1.30%)	44 (1.19%)
Filling	11 (1.19%)	163 (5.89%)	174 (4.69%)
Mean DMFT^#^	4.87 ± 4.36	6.92 ± 4.09	6.71 ± 4.15
Mean PLI^#^	1.43 ± 0.82	1.08 ± 0.68	1.18 ± 0.74
**ICDAS classification^**			
1–2 (Superficial)	-	-	494 (62.59%)
3–4 (Intermediate)	-	-	164 (20.79%)
5–6 (Deep)	-	-	131 (16.62%)

Notes ^, presented as range; #, presented as mean ± standard deviation; ^, total were the tooth surfaces with caries

**Table 2:** Caries situation in different tooth position and tooth surface

Variables	Number (%)	
	Sound	Caries
**Tooth position**		
Total	2793 (79.44%)	723 (20.56%)
Anterior tooth		
11–13	352 (89.80%)	40 (10.20%)
21–23	357 (91.54%)	33 (8.46%)
31–33	386 (97.23%)	11 (2.77%)
41–43	386 (97.23%)	11 (2.77%)
Premolar		
14–15	220 (87.30%)	32 (22.70%)
24–25	219 (86.56%)	34 (23.44%)
34–35	236 (91.47%)	22 (8.53%)
44–45	237 (91.86%)	21 (8.14%)
Molar		
16–17	125 (52.30%)	114 (47.70%)
26–27	127 (51.84%)	118 (48.16%)
36–37	76 (35.19%)	140 (64.81%)
46–47	73 (38.22%)	148 (61.78%)
**Tooth surface**		
Total	8689 (91.83%)	789 (8.17%)
Occlusal surface	1596 (76.07%)	502 (23.93%)
Buccal/Labial surface	3517 (95.31%)	173 (4.69%))
Lingual/Palatal surface	3576 (96.91%)	114 (3.09%)

**Fig. 3:**
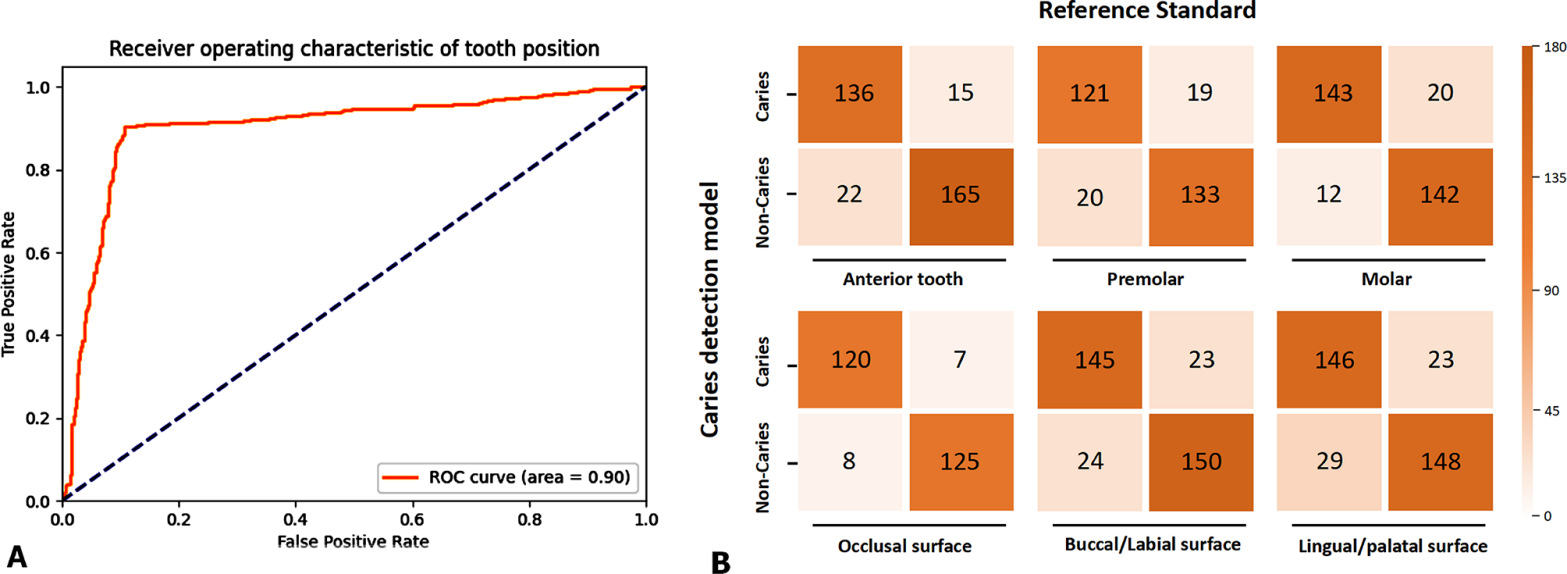
Performance of the QLF-based caries detection and staging model. Notes: curve 0, caries detection; curve 1, stage as ICDAS 1–2; curve 2, stage as ICDAS 3–4; curve 3, stage as ICDAS 5–6

A QLF-based caries detection and staging model was developed, and the model’s performance was satisfactory. Regarding caries detection ability, the validation cohort achieved an AUC of 0.88, ACC of 0.88, SPE of 0.94, SEN of 0.64, NPV of 0.90, PPV of 0.75, and F1 score of 0.69. (Fig. 3; Table 3). In the testing cohort, the overall accuracy was 0.92, with a sensitivity of 0.71 and a specificity of 0.95. When the model classified the caries into three stages, it performed best in deep caries detection (AUC of 0.84, ACC of 0.98, F1 score of 0.77), followed by intermediate caries detection (AUC of 0.90, ACC of 0.96, F1 score of 0.68) and superficial caries detection (AUC of 0.79, ACC of 0.90, F1 score of 0.63). In the testing cohort, the model still achieved an ACC between 0.93 and 0.99. (Table 4) Regarding the tooth position and surface, the diagnostic performance was best for molars, with an accuracy of 0.90, and for occlusal surfaces, with an accuracy of 0.94. The diagnostic performance of the model and the confusion matrix is shown in Table 3; Fig. 3. Typical QLF images and the detection performance are shown in Fig. 1.

**Table 3:** Performance of the QLF-based caries detection model in the validation and testing cohorts

Indicators	Validation cohort	Testing cohort								
		All	Tooth position				Tooth surface			
			Total	Anterior teeth	Premolar	Molar	Total	Occlusal surface	Buccal surface	Lingual surface
ACC	0.88 [0.86, 0.91]	0.92	0.87	0.89	0.87	0.90	0.88	0.94	0.86	0.85
SEN	0.64 [0.61, 0.69]	0.71	0.88	0.90	0.86	0.88	0.89	0.94	0.86	0.86
SPE	0.94 [0.93, 0.96]	0.95	0.89	0.88	0.87	0.92	0.87	0.94	0.86	0.84
NPV	0.90 [0.89, 0.92]	0.97	0.89	0.92	0.88	0.88	0.87	0.95	0.87	0.87
PPV	0.75 [0.72,0.78]	0.56	0.88	0.86	0.86	0.92	0.87	0.94	0.86	0.83
AUC	0.88 [0.86, 0.91]	-	-	-	-	-	-	-	-	-
F1 score	0.69 [0.67, 0.72]	0.62	-	-	-	-	-	-	-	-

*Notes* 95% confidence intervals are included in brackets. *Abbreviation* ACC, accuracy; SEN, sensitivity; SPE, specificity; NPV, negative predictive value; PPV, positive predictive value; AUC, area under the ROC curve

**Table 4:** Performance of the QLF-based caries staging model in the validation and testing cohorts

Indicators	Validation cohort			Testing cohort		
	Superficial caries (ICDAS 1–2)	Intermediate caries (ICDAS 3–4)	Deep caries (ICDAS 5–6)	Superficial caries (ICDAS 1–2)	Intermediate caries (ICDAS 3–4	Deep caries (ICDAS 5–6)
ACC	0.90 [0.88, 0.92]	0.96[0.94, 0.97]	0.98[0.96, 0.99]	0.93	0.98	0.99
SEN	0.58 [0.87, 0.94]	0.82[0.79, 0.85]	0.68[0.65, 0.69]	0.42	0.71	1.00
SPE	0.96 [0.87, 0.91]	0.97[0.96, 0.98]	0.99[0.97, 1.00]	0.97	0.98	0.99
NPV	0.93 [0.89, 0.92]	0.99[0.97, 1.00]	0.98[0.95, 0.99]	0.96	0.99	1.00
PPV	0.68 [0.87,0.92]	0.57[0.55, 0.58]	0.77[0.75, 0.79]	0.48	0.11	0.55
AUC	0.79 [0.89, 0.92]	0.90[0.88,0.92]	0.84[0.81, 0.86]	-	-	-
F1 score	0.63 [0.87, 0.92]	0.68[0.65, 0.70]	0.72[0.70, 0.75]	-	-	-

*Notes* 95% confidence intervals are included in brackets. *Abbreviation* ACC, accuracy; SEN, sensitivity; SPE, specificity; NPV, negative predictive value; PPV, positive predictive value; AUC, area under the ROC curve

## Discussion

The detection and diagnosis of dental caries are usually performed by a general dentist during a routine clinical examination, and most of the time, caries have advanced to a certain stage. The later the detection and treatment of caries are, the larger the tissue defect and the more complex the treatment. Since the early detection of caries through visual inspection is difficult [17] and people’s self-awareness is lacking, developing new methods to detect caries quickly and easily in daily life is lacking, such as a detector for home use. We developed a CNN model based on intraoral QLF images by a handheld device to intelligently and instantly detect and stage caries with excellent performance. The model can automatically identify decays without interpretation by a dentist, providing a convenient solution for people with insufficient motivation behind dental care and making at-home oral health testing possible. Compared to previous studies on AI caries detection [18–22], we used a relatively large dataset and adopted the QLF technology, which is currently recognized as effective in detecting tooth caries, especially in the early stage [23, 24], making our study reliable. Through this approach, our study not only achieved greater reliability but also provided dental institutions with an effective auxiliary diagnostic tool. Particularly for young dentists, this technology aids in caries detection, contributing to improving the diagnostic accuracy. This, in turn, mitigates the risk of misdiagnosis and consequently provides the overall precise oral health status for patients.

Currently, QLF has been used for detecting various diseases, such as dental caries, secondary caries [25], cracked teeth [26], tooth discolouration [27], and dentin-exposed tooth wear [28]. The QLF is an optical method that has good performance for the objective assessment of early changes in tooth enamel [29]. As one of the early caries detection methods, the greatest advantage of QLF technology is its high sensitivity [30]. In addition, the QLF enables noninvasive and nonradioactive multiple measurements and thus numerical, longitudinal monitoring of caries progression [31, 32]. When detecting caries, QLF can also detect red fluorescence, which is emitted by the porphyrin derivatives of bacterial metabolism [32]. Areas with greater aggregation of microorganisms, such as carious lesions, dental plaque, and dental calculus, will show increased red fluorescence. Therefore, AI equipment with QLF technology can realize convenient detection of caries at home and indicate the risk of caries by monitoring dental plaque in daily life to guide oral health care. However, QLF technology has limitations in detecting proximal caries [33] and is still subject to some factors that can lead to false positives, such as extrinsic stains [34]. Nevertheless, none of the above is sufficient to make the powerful combination of the QLF and AI less promising.

AI, especially deep learning, can identify the features of various image data and perform as well or better than human experts in caries diagnosis at various stages from images. In recent studies using CNNs for caries diagnosis, the images utilized included bitewing radiographs, CBCT, intraoral photographs, near-infrared light transillumination images, and periapical radiographs [35]. In these studies, the diagnostic accuracy of dental caries was similar to that of dentists, with the accuracy ranging between 0.82 and 0.89. Although X-ray technology, such as periapical or bitewing film, has a high sensitivity for proximal caries diagnosis, its ability to facilitate early caries diagnosis is limited. Studies using CT as an input signal can capture early enamel demineralization; however, the detection process is complicated [36]. The use of CNNs to assess dental caries in intraoral photographs is relatively uncommon. Typically, such assessments require thorough cleaning and drying of the tooth surfaces beforehand [12] and often necessitate the exclusion of various restorative materials or other concurrent hard tissue diseases before images can be captured for evaluation [37]. In contrast to these studies, our trained model does not require special preprocessing of the tooth surfaces and can be utilized by nonprofessional users to conduct the assessment. Additionally, because we annotated each image at the pixel level for carious regions, our model can provide users with the precise locations of suspected caries in the captured dental photographs. To achieve efficient diagnosis of dental caries, our study employed YOLOv5s as the training model. YOLOv5s is renowned for its highly flexible object detection performance [38], ability to train on large-scale datasets and ability to achieve rapid and accurate detection in real-time applications [14]. The selection of this model is attributed to its outstanding performance in general object detection tasks and sensitivity to fine-grained image features and adaptability to medical image processing [16]. The highly tuneable parameters and adaptability to diverse datasets inherent to YOLOv5s make it an ideal choice for this study, providing robust technical support for automating dental caries diagnosis. In this study, intraoral images after blue‒violet light excitation were used as input data; the overall accuracy was 0.88, which was higher than that of other similar studies.

Although the device we developed can accurately detect and stage caries lesions, some limitations remain in this study. First, the labels used in this study were not based on pathology but on the doctor’s examination by vision and a probe. To develop a more specific multiclass classifier according to degree, we will combine the X-ray film with a larger dataset in future studies. Second, the camera in the device does not have a high resolution. Because the mechanism of caries detection we used is based on the QLF, not the white light, fluorescence can compensate for the quality of the image used for AI development. Moreover, the cost is relatively low compared to that of commercial products, and the model performance was satisfactory.

## Conclusion

The caries detection and staging model based on the QLF portable device with a CNN can accurately detect caries lesions, which is expected to assist doctors in their diagnosis and increase oral health awareness of people through home use.

### Electronic supplementary material

Below is the link to the electronic supplementary material.

Supplementary Material 1

## Data Availability

The datasets generated and/or analysed during the current study are not publicly available due to protect the privacy of study participants but are available from the corresponding author on reasonable request.

## References

[1] Shivakumar K, Prasad S, Chandu G (2009). International Caries Detection and Assessment System: a new paradigm in detection of dental caries. J Conserv Dent.

[2] Janjic Rankovic M, Kapor S, Khazaei Y, Crispin A, Schüler I, Krause F, Ekstrand K, Michou S, Eggmann F, Lussi A (2021). Systematic review and meta-analysis of diagnostic studies of proximal surface caries. Clin Oral Investig.

[3] Macey R, Walsh T, Riley P, Glenny AM, Worthington HV, O’Malley L, Clarkson JE, Ricketts D (2021). Visual or visual-tactile examination to detect and inform the diagnosis of enamel caries. Cochrane Database Syst Rev.

[4] Sardana D, Ekambaram M, Yang Y, McGrath CP, Yiu CKY (2023). Caries-preventive effectiveness of two different fluoride varnishes: a randomised clinical trial in patients with multi-bracketed fixed orthodontic appliances. Int J Paediatr Dent.

[5] Liang JP (2021). [Research and application of new techniques for early diagnosis of caries]. Zhonghua Kou Qiang Yi Xue Za Zhi.

[6] Macey R, Walsh T, Riley P, Glenny AM, Worthington HV, Clarkson JE, Ricketts D (2021). Electrical conductance for the detection of dental caries. Cochrane Database Syst Rev.

[7] Zhang L, Sun T, Zhu P, Sun Z, Li S, Li F, Zhang Y, Tan K, Lu J, Yuan R (2020). Quantitative Analysis of Salivary Oral Bacteria Associated with severe early childhood caries and construction of Caries Assessment Model. Sci Rep.

[8] Sürme K, Kara NB, Yilmaz Y (2020). In Vitro Evaluation of Occlusal Caries Detection Methods in primary and permanent teeth: a comparison of CarieScan PRO, DIAGNOdent Pen, and DIAGNOcam methods. Photobiomodul Photomed Laser Surg.

[9] Zakian CM, Taylor AM, Ellwood RP, Pretty IA (2010). Occlusal caries detection by using thermal imaging. J Dent.

[10] Marmaneu-Menero A, Iranzo-Cortés JE, Almerich-Torres T, Ortolá-Síscar JC, Montiel-Company JM, Almerich-Silla JM. Diagnostic Validity of Digital Imaging Fiber-Optic Transillumination (DIFOTI) and Near-Infrared Light Transillumination (NILT) for caries in Dentine. J Clin Med 2020, 9(2). 10.3390/jcm9020420 PMC7073697 32033068

[11] Lee JW, Lee ES, Kim BI (2018). Optical diagnosis of dentin caries lesions using quantitative light-induced fluorescence technology. Photodiagnosis Photodyn Ther.

[12] Kühnisch J, Meyer O, Hesenius M, Hickel R, Gruhn V (2022). Caries Detection on Intraoral images using Artificial Intelligence. J Dent Res.

[13] Mohammad-Rahimi H, Motamedian SR, Pirayesh Z, Haiat A, Zahedrozegar S, Mahmoudinia E, Rohban MH, Krois J, Lee JH, Schwendicke F (2022). Deep learning in periodontology and oral implantology: a scoping review. J Periodontal Res.

[14] Bochkovskiy A, Chien-Yao W, Liao HYM. YOLOv4: optimal speed and accuracy of object detection arXiv. arXiv (USA) 2020:17 pp.-17 pp.

[15] Kaur R, Singh S (2023). A comprehensive review of object detection with deep learning. Digit Signal Prog.

[16] Jani M, Fayyad J, Al-Younes Y, Najjaran H. Model Compression Methods for YOLOv5: A Review. In.; 2023: arXiv:2307.11904.

[17] Macey R, Walsh T, Riley P, Glenny A-M, Worthington HV, Fee PA, Clarkson JE, Ricketts D (2020). Fluorescence devices for the detection of dental caries. Cochrane Database Syst Rev.

[18] Singh P, Sehgal P (2021). G.V Black dental caries classification and preparation technique using optimal CNN-LSTM classifier. Multimed Tools Appl.

[19] Wang C, Qin HT, Lai GY, Zheng G, Xiang HZ, Wang J, Zhang DW (2020). Automated classification of dual channel dental imaging of auto-fluorescence and white lightby convolutional neural networks. J Innov Opt Health Sci.

[20] Guijarro-Rodriguez AA, Witt-Rodriguez PM, Cevallos-Torres LJ, Contreras-Puco SF, Ortiz-Zambrano MC, Torres-Martinez DE. Image Segmentation techniques Application for the diagnosis of Dental Caries. Adv Emerg Trends Technol Adv Intell Syst Comput (AISC 1066) 2020:312–22.

[21] Holtkamp A, Elhennawy K, de Oro J, Krois J, Paris S, Schwendicke F (2021). Generalizability of Deep Learning models for Caries Detection in Near-Infrared Light Transillumination images. J Clin Med.

[22] Lakshmi MM, Chitra P. Classification of Dental Cavities from X-ray images using Deep CNN algorithm. 2020 *4th International Conference on Trends in Electronics and Informatics (ICOEI) Proceedings* 2020:774–779.

[23] Kim H-E, Kim B-I (2018). Early caries detection methods according to the depth of the lesion: an in vitro comparison. Photodiagn Photodyn Ther.

[24] Angmar-Månsson B, ten Bosch JJ (2001). Quantitative light-induced fluorescence (QLF): a method for assessment of incipient caries lesions. Dentomaxillofac Radiol.

[25] Brouwer F, Askar H, Paris S, Schwendicke F (2016). Detecting secondary caries lesions: a systematic review and Meta-analysis. J Dent Res.

[26] Jun M-K, Park S-W, Lee E-S, Kim B-R, Kim B-I (2019). Diagnosis and management of cracked tooth by quantitative light-induced fluorescence technology. Photodiagn Photodyn Ther.

[27] Min J-H, Kim B-R, Kim B-I (2021). Optical detection of the potential for tooth discoloration from children’s beverages by quantitative light-induced fluorescence technology. Photodiagn Photodyn Ther.

[28] Kim S-K, Jung HI, Kim B-I (2020). Detection of dentin-exposed occlusal/incisal tooth wear using quantitative light-induced fluorescence technology. J Dent.

[29] Maia AMA, de Freitas AZ, de Campello L, Gomes S, Karlsson ASL (2016). Evaluation of dental enamel caries assessment using quantitative light Induced fluorescence and Optical Coherence Tomography. J Biophotonics.

[30] Gomez J (2015). Detection and diagnosis of the early caries lesion. BMC Oral Health.

[31] Günther E, Park K-J, Meißner T, Kottmann T, Schmalz G, Haak R, Ziebolz D (2020). Assessment of non-cavitated root caries lesions by quantitative light-induced fluorescence-An in vivo feasibility study. Photodiagn Photodyn Ther.

[32] Cho KH, Kang C-M, Jung H-I, Lee H-S, Lee K, Lee TY, Song JS (2021). The diagnostic efficacy of quantitative light-induced fluorescence in detection of dental caries of primary teeth. J Dent.

[33] Oh SH, Choi J-Y, Kim S-H (2022). Evaluation of dental caries detection with quantitative light-induced fluorescence in comparison to different field of view devices. Sci Rep.

[34] Alammari MR, Smith PW, de Josselin de Jong E, Higham SM (2013). Quantitative light-induced fluorescence (QLF): a tool for early occlusal dental caries detection and supporting decision making in vivo. J Dent.

[35] Mohammad-Rahimi H, Motamedian SR, Rohban MH, Krois J, Uribe SE, Mahmoudinia E, Rokhshad R, Nadimi M, Schwendicke F (2022). Deep learning for caries detection: a systematic review. J Dent.

[36] Araki K, Matsuda Y, Seki K, Okano T (2010). Effect of computer assistance on observer performance of approximal caries diagnosis using intraoral digital radiography. Clin Oral Investig.

[37] Portella PD, de Oliveira LF, Ferreira MFC, Dias BC, de Souza JF, Assunção LRS (2023). Improving accuracy of early dental carious lesions detection using deep learning-based automated method. Clin Oral Invest.

[38] Redmon J, Farhadi A. YOLOv3: an incremental improvement. arXiv (USA); 2018.

